# Micro Satellite Orbital Boost by Electrodynamic Tethers

**DOI:** 10.3390/mi12080916

**Published:** 2021-07-31

**Authors:** Peter Yao, Timothy Sands

**Affiliations:** Sibley School of Mechanical and Aerospace Engineering, Cornell University, Ithaca, NY 14850, USA; py223@cornell.edu

**Keywords:** actuators, guidance, navigation, and control, cubesats, mini/micro satellites, spacecraft maneuvering, dynamics, magnetic field, tether, orbital dynamics, aerodynamic drag

## Abstract

In this manuscript, a method for maneuvering a spacecraft using electrically charged tethers is explored. The spacecraft’s velocity vector can be modified by interacting with Earth’s magnetic field. Through this method, a spacecraft can maintain an orbit indefinitely by reboosting without the constraint of limited propellant. The spacecraft-tether system dynamics in low Earth orbit are simulated to evaluate the effects of Lorentz force and torques on translational motion. With 500-meter tethers charged with a 1-amp current, a 100-kg spacecraft can gain 250 m of altitude in one orbit. By evaluating the combined effects of Lorenz force and the coupled effects of Lorentz torque propagation through Euler’s moment equation and Newton’s translational motion equations, the simulated spacecraft-tether system can orbit indefinitely at altitudes as low as 275 km. Through a rare evaluation of the nonlinear coupling of the six differential equations of motion, the one finding is that an electrodynamic tether can be used to maintain a spacecraft’s orbit height indefinitely for very low Earth orbits. However, the reboost maneuver is inefficient for high inclination orbits and has high electrical power requirement. To overcome greater aerodynamic drag at lower altitudes, longer tethers with higher power draw are required.

## 1. Introduction

Space, particularly in low-earth orbits, is littered with dangerous debris monitored by the National Aeronautics and Space Administration (NASA) who together with the United States Space Force issues warnings of impending space collisions [1]. Ecuador’s first and only satellite in orbit, Pegasus, collided with a Soviet-era rocket still in orbit [2]. In 2019 the European Space Agency spacecraft dodged potential collision with Starlink satellite when it maneuvered to avoid collision [3]. Collision avoidance is very challenging when the spacecraft at risk has no remaining fuel to perform maneuver, so this manuscript investigates the effectiveness of maneuvering spacecraft in low Earth orbit using electrodynamic tethers.

Spacecraft in low Earth orbit have a limited lifetime due to deceleration from aerodynamic drag that eventually cause low-earth orbiting satellites to re-enter (but on on-command). Reboost maneuvers can be conducted to keep them on station, however once the propellant is expended the spacecraft loses its maneuvering capability.

Spacecraft equipped with electrodynamic tethers are able to take advantage of Earth’s magnetic field and maneuver to avoid collision by generating miss-distance, or alternatively, reboost using electrical power, which can be generated from solar arrays. Such propellant-less maneuvers could extend spacecraft lifetime significantly; however, the effectiveness of maneuvering using electrodynamic tethers depends on several variables, including orbit eccentricity, inclination, altitude, and the charge and length of the tether. Various trajectories and tether configurations are illustrated via computer simulation investigating the functional limits of electrodynamic maneuvers in low Earth orbits. The proposed developments should prove especially useful for CubeSat whose extensive use of commercial off-the shelf components for their subsystems makes their cost a small fraction of the cost of traditional satellites, and thus likely ubiquitously lack propulsion systems to maintain low cost.

**Figure 1:**
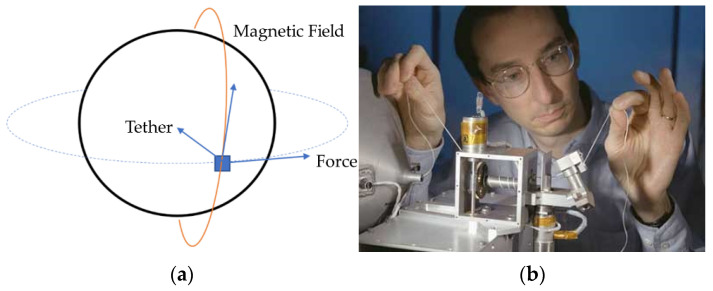
(**a**) Tether orientation for reboost. (**b**) Les Johnson, a scientist at Marshall Space Flight Center inspects nonconducting part of a tether [25].

Propellant-free space maneuvers [4] are currently a hot research topic usually performed by angular momentum storage devices such as reaction wheels or control moment gyroscopes [5]. The gyroscopes are complicated by singular mathematics [6], which was only recently solved [7]. Further complicating using momentum exchange devices is their limited capacity, and that capacity soon becomes saturated when they are required to absorb spacecraft disturbance torques caused by (aerodynamic drag [8], gravity gradient [9], solar wind [9], and electro-magnetic disturbances [10,11]). The proposals in this manuscript reverse the paradigm by instead seeking to utilize these external momentum source to intentionally modify spacecraft orbits, particularly by orienting an electric tether as depicted in Figure 1. Utilization of electric propulsion was recently proposed for ChipSats [12], and the dynamics were studied for CubeSats [13,14], including electric translational propulsion [15,16,17] in addition to tether-assisted propulsion [18] in the inaugural issue of *Aerospace*. This manuscript will study utilization of electric tethers to generate angular momentum, which propagates through the six coupled nonlinear equations of motion forming the basis for modern spacecraft attitude control and guidance proposed in 2007 [19], formulated in 2009 [20], and experimentally validated in 2012 [21]. The method will be evaluated for microsatellites. Electromagnetic tethers were recently proposed for spacecraft docking [22], and many other examples were just articulated in the review by O’Reilly et al., 2021 [23], which was already demonstrated by NASA to produce translational propulsion forces [24]. This manuscript will instead evaluate the resultant translational effects of coupled motion due to externally applied forces and torques through tethers. Multisatellite tether systems are not addressed.

Developments presented in this manuscript follow the nature of the 1998 NASA study [10], which focused on translational thrust generation using Lorentz force combined with the recent augmentation by Weis and Peck [12], which evaluated attitude control by Lorentz torques determined by Euler’s moment equations. Novelty is validated by the just published review [23] illustrating the assertion of the predominant focus of the literature on the generation of translational motion via thrust of the tether as a novel propulsion system or torque for attitude control.

This manuscript will add to the current state of the art by evaluation of the effects of Lorentz torque alone propagated through the full 66-term Euler’s moment equation and resultant coupled translational motion through the modification of angular velocity vector components appearing in Newton’s translational motion equations leading to translational motion.

While the coupling effects are nominally small (hundreds of meters), the evaluation illustrates useful propellant-free maneuvering capability not previously articulated by evaluation of the capability in five disparate sample low-earth orbits using simulations. The effect is also illustrated to be capable of changing orbital inclination tens of millidegrees (another small but non-negligible, propellant-free capability). A rare illustration of computation accuracy is offered foremost to illustrate the numerical precision of the simulation, which is included in Appendix B to aid repeatability.

## 2. Materials and Methods

### 2.1. Mechanics

According to Chasle [26], mechanical motion of rigid bodies may be fully articulated by invoking three equations from Newton’s second law [27] for translation and three equations from Euler’s equations [28] for rotation. Smeresky et al., (2019) present the nonsimplified rotation equations in [29], presented here as Equation (1) in two disparate compact vector-matrix notations, where Equation (4) displays the noncompact form that illuminates the propagation of angular velocity modifications through all three equations of rotational state motion.

(1) $$\sum \tau =J\dot{\omega }+\omega \times J\omega =\left[\Phi \right]\left\{\Theta \right\}\;$$

where vector and matrix components are defined in Table 1 elaborated in Equations (2)–(4), whose components are defined in Table 2.

Equation (1) reparametrizes the better-known formulation on the left of the equation into the lesser-known regression form on the right of the equation by defining a matrix of presumed “knowns” and a vector of parameters potentially to be estimated. The regression matrix is defined in Equation (2), while the regression vector is defined in Equation (3) with the variables’ definitions in Table 2. Here, a third parameterization is depicted in Equation (4), where all terms are of Equation (1) and are multiplied out revealing the nonlinear coupling upon quick inspection.

(2) $$\left[\Phi \right]=\left[\begin{array}{ccc} {\dot{\omega }}_{x} & {\dot{\omega }}_{y} & {\dot{\omega }}_{z}\\ \omega_{x}\omega_{z} & {\dot{\omega }}_{x} & 0\\ -\omega_{x}\omega_{y} & 0 & {\dot{\omega }}_{x} \end{array}\;\;\begin{array}{ccc} -\omega_{y}\omega_{z} & 0 & \omega_{z}\omega_{y}\\ {\dot{\omega }}_{y} & {\dot{\omega }}_{z} & -\omega_{z}\omega_{x}\\ \omega_{y}\omega_{x} & {\dot{\omega }}_{y} & {\dot{\omega }}_{z} \end{array}\right]\;$$

(3) $$\Theta ={\left\{J_{xx},\;J_{xy},J_{xz},J_{yy},J_{yz},J_{zz}\right\}}^{T}$$

(4) $$\left\{\begin{array}{c} \tau_{x}\\ \tau_{y}\\ \tau_{z} \end{array}\right\}=\{\begin{array}{c} J_{xx}{\dot{\omega }}_{x}+J_{xy}{\dot{\omega }}_{y}+J_{xz}{\dot{\omega }}_{z}-J_{xy}\omega_{x}\omega_{z}-J_{yy}\omega_{y}\omega_{z}-J_{yz}\omega_{z}^{2}+J_{xz}\omega_{x}\omega_{y}+J_{zz}\omega_{z}\omega_{y}+J_{yz}\omega_{y}^{2}\\ J_{yx}{\dot{\omega }}_{x}+J_{yy}{\dot{\omega }}_{y}+J_{yz}{\dot{\omega }}_{z}-J_{yz}\omega_{x}\omega_{y}-J_{zz}\omega_{x}\omega_{z}-J_{xz}\omega_{x}^{2}+J_{xx}\omega_{x}\omega_{z}+J_{xy}\omega_{z}\omega_{y}+J_{xz}\omega_{z}^{2}\\ J_{zx}{\dot{\omega }}_{x}+J_{zy}{\dot{\omega }}_{y}+J_{zz}{\dot{\omega }}_{z}-J_{xx}\omega_{x}\omega_{y}-J_{xz}\omega_{y}\omega_{z}-J_{xy}\omega_{y}^{2}+J_{yy}\omega_{x}\omega_{y}+J_{yz}\omega_{z}\omega_{x}+J_{xy}\omega_{x}^{2} \end{array}\}$$

(5) $$\sum F=ma=\left[m\right]\left\{a_{r\;}-\left(2\omega \times \dot{r}\right)-\omega \times \left(\omega \times r\right)-\dot{\omega }\times r\right\}$$

Meanwhile Equation (5), whose components are defined in Table 3, elaborates how the external application of forces produces translation motion; the equation is also coupled to Equation (4)’s rotational motion through the angular velocity, ω. Equation (4), whose components are defined in Table 3, clearly illustrates how a change in a single angular momentum component $J_{ij}\omega_{k}$ results in motion throughout all three rotational equations in Equation (4). Similarly, Newton’s Law expressed in rotating reference frames results in three coupled nonlinear equations that also include cross-products with angular velocity, ω Equation (5). This coupling is well-known in all avenues of motion mechanics, even ocean vehicles (see the opening equations in [30]). Modification of a component of angular momentum in Equation (1) or Equation (4) modifies the respective component of angular velocity [which resides in Equation (5) as well]. Thus, the identical angular momentum component in Equation (5) is modified, propagating through all three translational motion equations, as was the case with all three equations of rotational motion. Translational velocity components are modified as linear momentum (which remains conserved) swaps between the three channels of translational motion.

### 2.2. External Forces and Torques, F and τ

**Figure 2:**
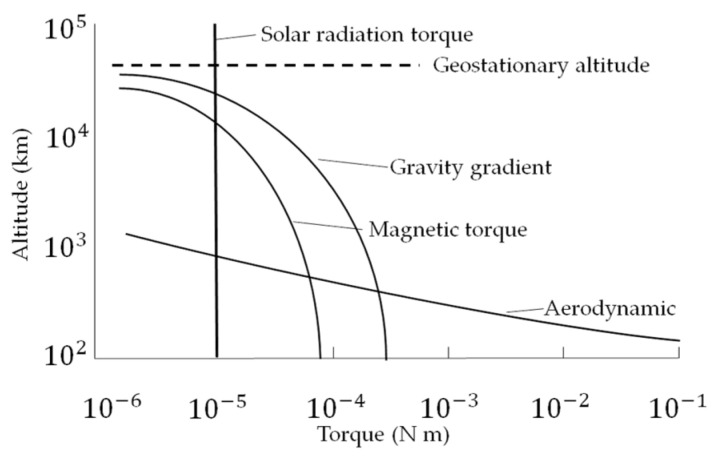
Rough order-of-magnitude of environmental torques available to spacecraft as a function of altitude. These environmental torques may be used for angular momentum modification, and this manuscript focuses on such modification using only magnetic torques.

Four of the main disturbance forces acting on Earth orbiting spacecraft (whose rough relative characteristics are displayed in Figure 2): gravity gradient, aerodynamic drag, and magnetic field forces are simulated (solar radiation pressure is omitted). Aerodynamic drag forces dominate in low earth orbits, while gravity gradient and then magnetic forces dominate at altitudes on the order of thousands of kilometers. Solar pressure forces in Equation (6) are four orders of magnitude smaller than aerodynamic forces at altitudes less than 500 km.

Environmental forces and torques are included in the validating simulation, while magnetic forces and torques are the focus of the investigation of useful production of translation. Aerodynamic drag force is calculated using Equation (6), where ρ is the atmospheric density from the (Mass Spectrometer-Incoherent Scatter) MSISE-90 atmosphere model [31,32] under mean solar conditions. No torque is produced from drag if the center of pressure and center of gravity are coincident. Gravity gradient torque elaborated in Equation (7) is a function of gravitational parameter *μ* [33,34], as reported by [35], orbit altitude *R*, moment of inertia matrix *J*, and body frame. Magnetic torque caused by Earth’s magnetic field is represented in Equation (8). This torque depends on the magnetic moment *M_M_* and magnetic field *B*. Variables for Equations (6)–(8) are defined in Table 4.

(6) $$F_{drag}=-\frac{1}{2}C_{D}A\rho v^{2}$$

(7) $$\tau_{g}=3\frac{\mu }{R^{3}}J\times \delta_{B}$$

(8) $$\tau_{M}=M_{M}\times B$$

Although aerodynamic disturbances have greater influence than magnetic disturbances in low Earth orbits, magnetic effects can still be used to maneuver a spacecraft. By interacting with Earth’s magnetic field, rotational and translational motion can be produced without expending propellant. Magnetorquers create magnetic dipoles which exert a force on the surrounding magnetic field at moment arms described in Equation (8). A magnetorquer with vector area (*A*) and current (*I*) passing through *n* coils will generate a torque that can rotate a spacecraft. Similarly, an electrically charged tether can produce translational motion using the Lorentz force in Equation (9). Based on the orientation of the tether (depicted in Figure 1a), its interaction with the local magnetic field can provide a boosting or deorbiting force. Several missions demonstrated application of electrodynamic tethers, and the principle was proposed for use on the International Space Station [10]. A unique contribution here is the analysis of utilization of electromagnet Lorenz torques modifying angular momentum components in accordance with Equation (4), with resulting propagation though translation motion described in Equation (5) without modifying velocity magnitude. Variables for Equations (9) and (10) are defined in Table 4.

(9) $$\tau_{Lorentz}=nIA\times B$$

(10) $$F_{Lorentz}=\int_{0}^{L}I\left(L\right)dL\times B$$

A magnetic field will exert a force on a current carrying wire in a direction perpendicular to the wire and the field vector. By orienting a tether along the gravity vector towards Earth, the resulting force will be aligned with the spacecraft’s velocity vector. The resulting acceleration will boost the spacecraft to a higher orbit and counteract the deceleration caused by aerodynamic drag.

Not all orbits are ideal for electrodynamic maneuvering. The strength of the magnetic field will vary depending on the spacecraft’s eccentricity, inclination, and altitude. Circular equatorial orbits at various altitudes are simulated first to understand the best-case scenario performance of an electrodynamic tether reboost. Orbit inclination and tether deflection angle are additional independent variables that will be investigated to test the effectiveness of the maneuver. Tether deflection can be caused by aerodynamic drag. When deployed at low altitudes, the tether will deflect behind the spacecraft if there is insufficient end mass to keep the tether taut. This deflection can reduce reboost efficiency. To increase energy efficiency, current (*I*) can be reduced by proportionately increasing tether length (*L*) to produce an equivalent Lorentz force. Tethers of varying lengths and charge are simulated to determine the limits of electrodynamic maneuvering efficiency.

### 2.3. Spacecraft Simulation

In the simulation, a 1 m^3^ microsatellite with a mass of 100 kg is deployed in a low Earth orbit between 100 km and 500 km altitude with electrodynamic tethers. This test spacecraft has a coefficient drag of 2.2, which is a typical value for satellite simulations [8]. It is assumed that the satellite’s center of pressure is coincident with the center of gravity.

The tether system is assumed to have sufficient conductivity to maintain a one ampere current through lengths of 500 m as well as a low mass density. Historical tether designs have typically used a copper conductor with Kevlar insulation, such as the tether used for the TSS-1R tether experiment flown aboard STS-75. A detailed investigation of the power systems used to maintain the current through the tether system is out of the scope of this manuscript. However, power consumption is a major factor in the effectiveness of this maneuver technique.

### 2.4. Validating Simulations

To validate the assertion that Lorentz torques will propagate through all six coupled Equations of motion in Equations (4) and (5), simulation models were created and run in MATLAB/SIMULINK R2021a. Depictions of the simulations are included in Appendix B to aid repeatability along with accompanying MATLAB code and the results are described in Section 3.

### 2.5. Simulation Accuracy

To verify that the simulation is producing accurate results, the numerical solver and step size was chosen to limit the numerical error. The maximum precision obtainable is called machine precision for floating point arithmetic. As the quaternion normalization necessitates unity normalization, the difference between the calculated normalized quaternion and unity is utilized to represent the numerical accuracy of the simulation with various simulation step sizes and solver properties. Several solver and step size combinations are investigated to determine an option that produces accurate results, where $10^{-15}$ is used as an accuracy standard in this study.

## 3. Results

After presenting simulations performed to validate accuracy in Section 3.1, reboost by electrodynamic tether is presented in Section 3.2 before discussing the results in broad terms in Section 4.

### 3.1. Verification of Simulation Accuracy

**Figure 3:**
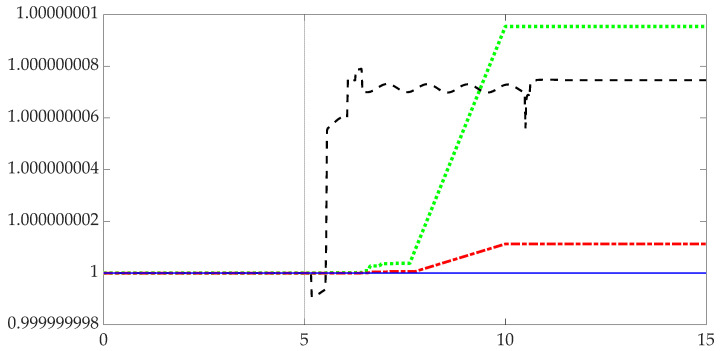
Comparison of quaternion normalizations (on the ordinate) for different step sizes using the Runge–Kutta solver. Standard deviations for 50 (black dashed line), 250 (green dotted line), 500 (red dot-dashed line), and 1,000 Hertz (blue solid thin line) sample rates are displayed. Time in seconds is displayed on the abscissa with quaternion normalization on ordinate.

This section illustrated a well-known adage: computer simulations can seemingly be made to indicate very different things. Visual inspection of Figure 3 reveals four identical simulation calculations with immediately obvious differences in results driven here (in this manuscript) by both the integration solver selected and the discretization time interval or step-size. Figure 3 illustrates the step-size alone results in disparate accuracies assuming the same integration solver. Table 5 indicates the results of iterating four different integration solvers and down-selecting to the Runge–Kutta solver with subsequent integration of four step-sizes eventually selecting 0.001 s with a numerical precision calculating the quaternion normalization condition of $5.0\times 10^{-15}$ (standard deviation).

Utilizing the most accurate integration solver and step-size iterated in Section 3.1, Section 3.2 describes the ability of using electromagnetic tethers to modify angular momentum (through modified angular velocity), and the modified angular velocities are seen to propagate through all six Equations (4) and (5) cyclically boosting the orbital attitude of the microsatellite.

### 3.2. Performance of Electrodynamic Tether Reboost

A microsatellite of 1 m^3^ size was simulated deploying 500-m tether system charged to 1 amp. The total mass of the spacecraft is 100 kg. All data presented in this manuscript use the same spacecraft model. The simulations are used to iterate initial orbit parameters and tether deflection angle.

**Figure 4:**
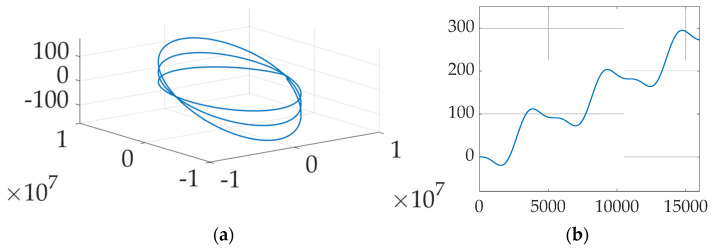
Effect of electrodynamic tether reboost on a 300 km altitude orbit. (**a**) Trajectory. (**b**) Altitude change in meters on ordinate versus time on abscissa.

**Figure 5:**
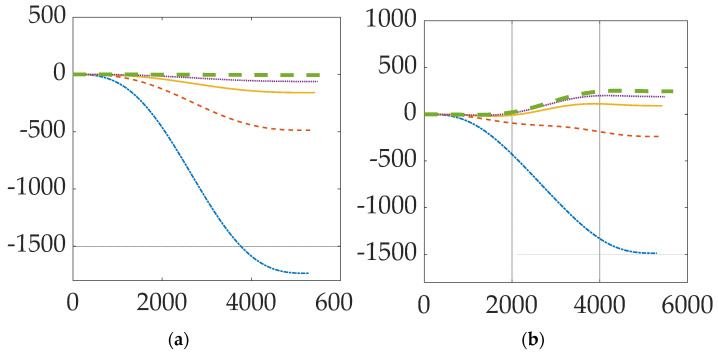
Altitude-change over one orbit with time in seconds on the abscissa and altitude change in meters on the ordinate. 200 km is blue, dash-dotted line; 250 km is red dashed line; 300 km is solid yellow line; 350 km is dotted purple line, and 500 km is green, thick dashed line: (**a**) Natural orbit decay from disturbances; (**b**) results with tether deployed.

**Figure 6:**
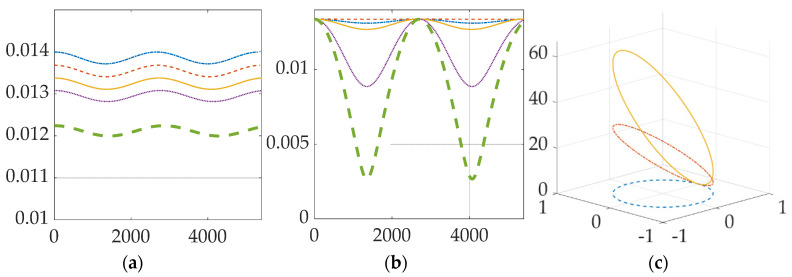
Lorentz force extracted from electrodynamic tether with time in seconds on the abscissa and (**a**) Altitude (kilometers) on the ordinate where 200 km is blue, dash-dotted line; 250 km is red dashed line; 300 km is solid yellow line; 350 km is dotted purple line, and 500 km is green, thick dashed line; (**b**) Inclination (degrees) on the ordinate, respectively where 0 degrees is blue, dash-dotted line; 10 degrees is red dashed line; 30 degrees is solid yellow line; 60 degrees is dotted purple line, and 90 degrees is green, thick dashed line. (**c**) Lateral miss distance (meters) from orbit trajectory due to tether deflection (degrees) over one revolution on the ordinate. Zero degrees is displayed by the dashed blue; 10 degrees is displayed by the red dashed line; 20 degrees is displayed by the solid yellow line. $\;\hat{x}$ and $\;\hat{y}$ coordinates ($\times 10^{7}$ ) on the horizontal plane with $\;\hat{z}$ coordinates displayed vertically.

Figure 4 displays the natural orbit decay and the boost height gained. Figure 5 shows the change in orbit trajectory and absolute change in altitude over a period of three orbits. Figure 6 displays the periodic variance in Lorentz force obtained from the electrodynamic tether and the effects of altitude and inclination. Both Figure 5 and Figure 6 depict a spacecraft flying a circular equatorial orbit, except for Figure 6b which depicts 300 km orbits of varying inclination. Table 6 presents numerical values for the data shown graphically in Figure 6. Atmospheric density is assumed to be constant; the atmospheric density at the initial altitude is used throughout each simulation.

Table 7 gives numerical Tabls for means and standard deviations of Lorentz force produced by the electrodynamic tether in orbits of varying inclinations. Figure 6 describes the effects of tether deflection on reboost maneuver efficiency. Figure 6a shows the absolute altitude gain with respect to the center of the Earth. Figure 6b compares the change in force produced by the tether due to the deflection. Figure 6c is a visual representation of the off-axis movement in the orbital trajectory caused by tether deflection.

With these results (tabularized and depicted for ease of reading), the next section describes the results in a narrative including how they can be interpreted from the perspective of previous studies and of the working hypotheses. The findings and their implications are discussed in the broadest context possible, and future research directions are also be highlighted.

## 4. Discussion

Table 5 and Figure 3 indicate the solver scheme declared to produce sufficient accuracy for validating simulations is the Runge–Kutta solver when used with a step size of 1 ms. As no other solver schemes produced accurate mean normalized quaternions, those other solver and step-size combinations are declared insufficiently accurate.

Figure 4 shows a periodic increase in altitude of 91,112 m per orbit starting at three-hundred-kilometers following electrodynamic tether system activation. Orbit altitude temporarily decreases every orbit period; however, the net change is still a positive increase in altitude. Slight decreases in altitude occur regularly and can be observed at the one-quarter and three-quarter points in each orbit cycle. Over three orbits, the cross-track deviation in spacecraft position is 100 m left and right. Increase in orbit inclination can be attributed to cross track velocity (component) changes caused by the Lorentz torque. This can be minimized by actively controlling the tether direction.

Figure 5a shows the effect of aerodynamic drag forces on spacecraft orbiting at low altitudes. Lower orbits will have greater altitude loss due to increased aerodynamic drag. Table 6 shows that over one orbit period, a 200 km altitude orbit will drop by 1776 m, while a 500 km altitude orbit will drop by only 5 m. Over multiple orbits, altitude losses will compound exponentially. This can be observed with the 200 km altitude spacecraft, which experiences the greatest altitude loss in one orbit. Although altitude losses for spacecraft with higher orbits are not as significant, over multiple orbits the altitude changes will compound as well. Overall, the life expectancy of such spacecraft without reboost options are on the order of hours to days. Figure 5b shows the altitude change from the reboost maneuver. Figure 5b appears to show that the electrodynamic reboost maneuver is more effective at higher altitudes, however this is not the case. The greater altitude gain recovered by the maneuver is due to the decrease in atmospheric density, which results in lower aerodynamic drag force. However, periodic variations are observed in altitude gain due to cross-track velocity changes from Lorentz torque. Greater altitude gain can be observed between the one quarter and three-quarter points in each orbit cycle. Table 6 confirms that the altitude gain is nearly constant at 249.8 m. Slight changes are caused by variations in magnetic field strength at different altitudes. Extrapolation from Table 6 data implies that around an altitude of 275 km, the spacecraft could achieve a state of equilibrium by balancing Lorentz force with aerodynamic drag.

Figure 6a confirms that as altitude increases, the Lorentz force decreases as well due to the weakening of the magnetic field. However, altitude gain increases as altitude increases, as displayed in Table 6, illustrating that less force is required to gain altitude in higher orbits due to the decrease in atmospheric density.

Figure 6b plots the periodic variation in Lorentz force throughout one orbit period. The periodic variation is due to the magnetic field lines travelling at an angle not perpendicular to the circular equatorial orbit of the spacecraft. Earth’s magnetic axis is not parallel to its rotation axis. Every year the magnetic poles shift. As of the year 2020, the epsilon angle between Earth’s magnetic and rotation axes is 11 degrees. When the inclination of the orbit was changed to 10 degrees in Figure 6b, the periodic variation of Lorentz force decreased as the spacecraft’s orbit was nearly perpendicular to the magnetic field lines. In this condition, the generated force was near the maximum during the entire orbit. Higher inclinations experience greater variations in force generation due to tether position with respect to the magnetic field lines. Force output decreases sinusoidally as the spacecraft’s distance to the magnetic plane increases. Therefore, the effectiveness of the electrodynamic tether reboost maneuver decreases as the inclination of the spacecraft’s orbit deviates from the epsilon angle. Table 6 gives numerical means and standard deviations showing the variance in force generation. The mean force generated is greatest and had the least variance when the orbit and magnetic axes were aligned.

Figure 6c shows tether deflection causes the spacecraft to deflect laterally along its orbit trajectory. Lateral deflections on the order of 100 m are expected when the tether is deflected up to 20 degrees. Tether orientation can be adjusted such that maneuvering in alternate directions is possible. Due to the orientation of the magnetic axis, the reboost maneuver will cause the orbit inclination to increase. This can be countered by reorienting the tether, but this also adds the necessity of an active control framework for the tether. The lateral motion generated from tether deflection also opens the possibility of multidirectional maneuvering using electrodynamic tethers, where a tether directional control system could be manipulated to generate vectored thrust.

## 5. Conclusions

In this manuscript, the dynamics of orbital boost maneuvers for microsatellites using electrodynamic tethers is investigated. This manuscript offers a novel contribution by evaluating the effects of both Lorentz force and Lorentz torque propagation through the full sixty-six term Euler’s moment equation and resultant coupled translational motion through the modification of angular velocity vector components appearing in Newton’s translational motion equations. Computational accuracy is also provided to demonstrate the numerical precision of the simulation.

Electrodynamic tethers are a viable instrument for reboosting satellites in low Earth orbit, provided that sufficient power generation is available. Mass and budget savings from eliminating the need for expendable propellant can save billions of dollars over a 10-year period [3]. Smaller satellites equipped with small tethers only 500 m in length and a 1 amp current would be able to operate at low altitudes for longer times. The spacecraft could conserve power by running current through the tether during portions of the orbit that would produce maximum Lorentz force. By evaluating the effects of both Lorentz force and Lorentz torque propagation through Euler’s moment equation and Newton’s translational motion equations, the simulated spacecraft-tether system can orbit indefinitely at altitudes as low as 275 km.

Further investigation of the effectiveness of the propellant-free reboost using electrodynamic tethers will require a study of the power requirements of the tether system and detailed design of the system. Limitations introduced by the power generation capabilities of microsatellites will reduce the effectiveness of the reboost maneuver. Mechanical properties of the tether must also be modelled in greater detail to understand deployment, lifetime analysis, and libration modes.

Following these results illustrating the ability to generate propellant-free translational maneuvers by modifying the angular velocity with rotational maneuvers utilizing energy from the Earth’s magnetic field, future research will focus on methods to generate predicted maneuvers to command generating specified translational maneuvers. The previously cited [30] elaborates such a method applied to unmanned underwater vehicles, and the next directions of research will develop applications of deterministic artificial intelligence to autonomous spacecraft utilizing electrodynamic tethers in accordance with this manuscript.

## Figures and Tables

**Table 1:** Definitions of variables proximal to Table 1.

Variable	Definition	Variable	Definition	Variable	Definition	Variable	Definition
J	mass moment of inertia	τ	applied torque	${\dot{\omega }}_{x}$	Angular acceleration about x-direction	$\omega_{x}$	Angular velocity about x-direction
$\dot{\omega }$	angular acceleration	Φ	regression matrix	${\dot{\omega }}_{y}$	Angular acceleration about y-direction	$\omega_{y}$	Angular velocity about y-direction
ω	angular velocity	Θ	regression vector	${\dot{\omega }}_{z}$	Angular acceleration about z-direction	$\omega_{z}$	Angular velocity about z-direction

**Table 2:** Definitions of variables proximal to the Table 2.

Variable	Definition	Variable	Definition
$J_{xx}$	mass moment of inertia with respect to the *x*-axis	$J_{xy}$	mass product of inertia–sum of the products formed by multiplying each element of mass by the product of the *x* and *y* coordinates
$J_{zz}$	mass moment of inertia with respect to the *z*-axis	$J_{yz}$	mass product of inertia–sum of the products formed by multiplying each element of mass by the product of the *y* and *z* coordinates
$J_{yy}$	mass moment of inertia with respect to the *y*-axis	$J_{xz}$	mass product of inertia–sum of the products formed by multiplying each element of mass by the product of the *x* and *z* coordinates

**Table 3:** Definitions of variables proximal to Table 3.

Variable	Definition	Variable	Definition	Variable	Definition
$\tau_{x}$	applied torque about x-direction	F	applied force	$\dot{r}$	displacement rate relative to rotating frame
$\tau_{y}$	applied torque about y-direction	m	mass	r	displacement relative to rotating frame
$\tau_{z}$	applied torque about z-direction	a	acceleration	$a_{r\;}$	apparent acceleration

**Table 4:** Definitions of variables proximal to Table 4.

Variable	Definition	Variable	Definition
ρ	Atmospheric density	$\tau_{Lorentz}$	Lorentz torque
*µ*	Standard gravitational parameter	n	Number of coils
*R*	Earth radius	*A*	Area
*M_I_*	Magnetic moment	*I*	Current
L	Tether length	*B*	Magnetic Field Vector
dL	Tether length differential	$F_{Lorentz}$	Lorentz Force

**Table 5:** Quaternion normalization errors for different solver schemes ^1^.

Solver	Step Size	Mean	Standard Deviation
Euler (ode1)	0.001	$2.5\times 10^{-5}$	$2.2\times 10^{-5}$
Heun (ode2)	0.001	$6.5\times 10^{-11}$	$6.1\times 10^{-11}$
Dormand–Prince (ode5)	0.001	$2.0\times 10^{-12}$	$1.2\times 10^{-12}$
Runge–Kutta (ode4)	0.020	$2.0\times 10^{-9}$	$3.5\times 10^{-9}$
Runge–Kutta (ode4)	0.010	$3.9\times 10^{-9}$	$4.4\times 10^{-9}$
Runge–Kutta (ode4)	0.002	$4.7\times 10^{-10}$	$5.2\times 10^{-10}$
Runge–Kutta (ode4)	0.001	$1.0\times 10^{-15}$	$5.0\times 10^{-15}$

^1^ Runge–Kutta (ode4) with 0.001 step-size was selected.

**Table 6:** Orbit and altitude parameters for implementation at various initial altitudes.

Initial Altitude (km)	Orbit Period (s)	Final Altitude without Tether (km)	Final Altitude with Tether (km)	Altitude Gain from Tether (m)
200	5301.245	198.264	198.513	249.680
250	5361.245	249.513	249.763	249.796
300	5422.715	299.841	300.091	249.828
350	5483.790	349.939	350.189	249.838
500	5668.390	499.995	500.245	249.848

**Table 7:** Means and standard deviations of Lorentz force at varying inclinations.

Inclination (°)	Mean Force (*N*)	Standard Deviation
0	0.01324	9.462776735569736 × 10^−5^
10	0.013374031380808	1.633511026861032 × 10^−6^
11.5	0.013376428353265	1.077122200582248 × 10^−7^
20	0.013374031380808	5.225155534655572 × 10^−5^
30	0.013034404952252	2.449844931947672 × 10^−4^
60	0.011209316951912	0.001576691931541
90	0.008873540981345	0.003581649585287

**Figure A1:**
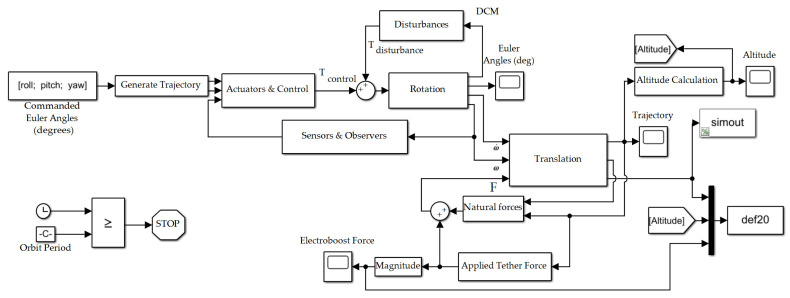
SIMULINK simulation created to validate proposals.

**Figure A2:**
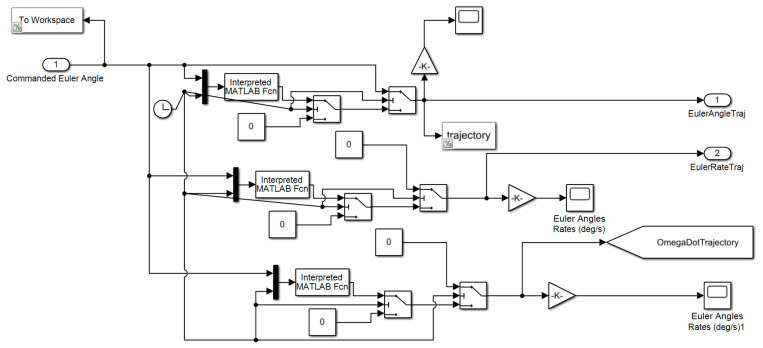
*Generate Trajectory* subsystem in Figure A1.

**Figure A3:**
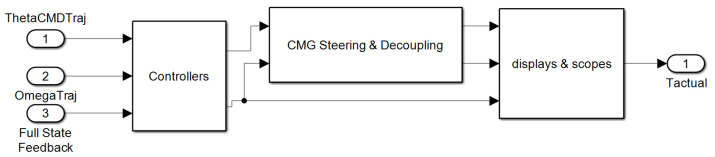
*Actuators and Control* subsystem in Figure A1.

**Figure A4:**
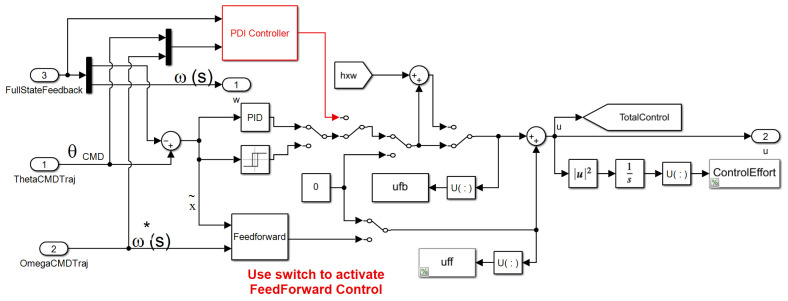
*Controllers* subsystem in Figure A3. The * indicates optimal values.

**Figure A5:**
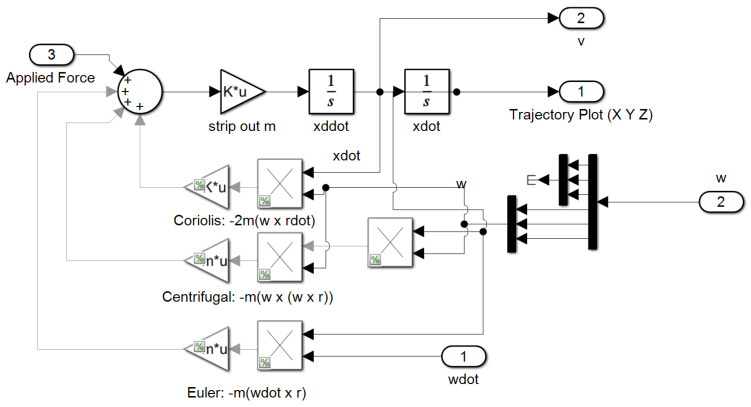
*Translation* subsystem in Figure A1.

**Figure A6:**
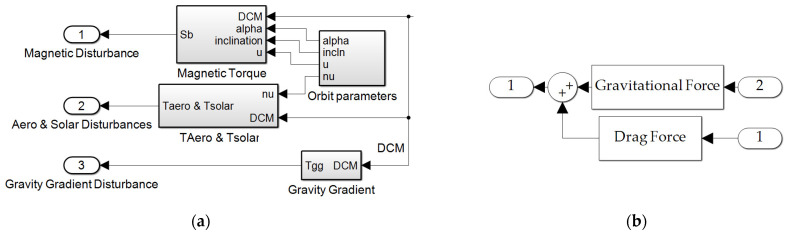
SIMULINK subsystems in Figure A1 (**a**) *Disturbances* subsystem; (**b**) *Translation* subsystem.

**Figure A7:**
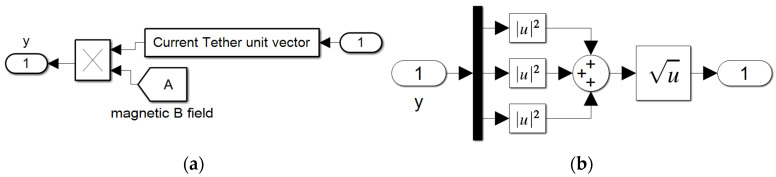
SIMULINK subsystems in Figure A1 (**a**) *Applied Tether Force* subsystem; (**b**) *Magnitude* subsystem.

**Figure A8:**
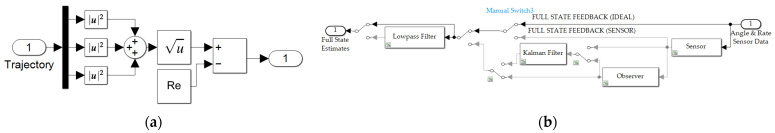
SIMULINK subsystems in Figure A1 (**a**) *Altitude Calculation* subsystem; (**b**) *Sensors and Observers* subsystem (bypassed).

**Figure A9:**
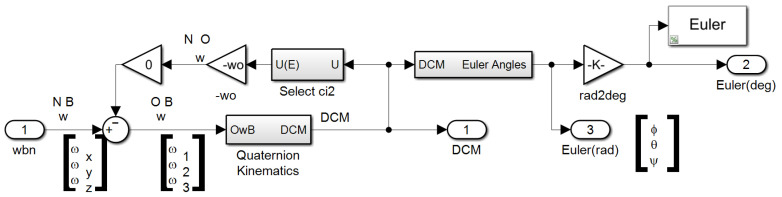
*Kinematics* subsystem in the *Rotation* block in Figure A1.

## Data Availability

Data supporting reported results can be found by contacting the author at py223@cornell.edu.

## Appendix A. Consolidated Variables and Acronyms

This appendix contains a table of variable and acronym definitions used in the manuscript.

**Table A1:** Variables and acronyms.

Variable/Acronym	Definition
J	mass moment of inertia
$\dot{\omega }$	angular acceleration
ω	angular velocity
τ	applied torque
Φ	regression matrix
Θ	regression vector
${\dot{\omega }}_{x}$	Angular acceleration about x-direction
${\dot{\omega }}_{y}$	Angular acceleration about y-direction
${\dot{\omega }}_{z}$	Angular acceleration about z-direction
$\omega_{x}$	Angular velocity about x-direction
$\omega_{y}$	Angular velocity about y-direction
$\omega_{z}$	Angular velocity about z-direction
$J_{xx}$	mass moment of inertia with respect to the *x*-axis
$J_{yy}$	mass moment of inertia with respect to the *y*-axis
$J_{zz}$	mass moment of inertia with respect to the *z*-axis
$J_{xy}$	mass product of inertia—sum of the products formed by multiplying each element of mass by the product of the *x* and *y* coordinates
$J_{yz}$	mass product of inertia—sum of the products formed by multiplying each element of mass by the product of the *y* and *z* coordinates
$J_{xz}$	mass product of inertia—sum of the products formed by multiplying each element of mass by the product of the *x* and *z* coordinates
$\tau_{x}$	applied torque about x-direction
$\tau_{y}$	applied torque about y-direction
$\tau_{z}$	applied torque about z-direction
F	applied force
m	mass
a	acceleration
$\dot{r}$	displacement rate relative to rotating frame
r	displacement relative to rotating frame
$a_{r\;}$	apparent acceleration
ρ	atmospheric density
*µ*	gravitational parameter
*R*	orbit altitude
L	tether length
dL	differential tether length
$\tau_{Lorentz}$	Lorentz torques
n	number of coils in magneto-torquer
*A*	magneto-torquer vector area
*I*	magneto-torquer current
*B*	Earth’s magnetic field
$F_{Lorentz}$	Lorentz forces

## Appendix B

The appendix contains SIMULINK models in figures and computer codes inserted into the *InifFcn* callback and *StopFcn* call back respectively. Together, this is the only computer code need to achieve the results presented in this manuscript.

**Algorithm A1.** InitFcn callbacks for SIMULINK model depicted in Figure 3, Figure 4, Figure 5 and Figure 6
% Simulation run parameters Rate = 34.3; DeltaT = 1/Rate; %Constants0 Re = 6371.2e3; mu = 398601.2e9; %earth radius and universal gravitation constant %Spacecraft orbit R = Re + h; %orbit radius from center of earth we = 0.000072921158553; %earth’s angular velocity rad/solar sec(Vallado) wo = sqrt(mu/(R)^3); %orbit angular velocity epsilon = 11.5*pi/180; %angle between magnetic and geographic pole axes alphao = 0; uo = 0; nuo = 0; %Start S/C beneath subsolar point betasun = 60; gamma = 1.5; a = 1; b = 1; c = 1; %Assumed spacecraft rectangular size Area = [b*c a*c a*b]; %projected area~m^2 in body x, y, z directions kpre = −9.9639/24/3600/180*pi*0; %nodal precession constant assumed zero here wn = kpre*(Re/(Re + h))^3.5*cos(incln); %nodal precession (zero eccentricity) V = wo*(Re + h); rho = asin(Re/(h + Re)); %earth angular radius psun = 4.5E-6; %solar pressure constant~N/m^2 NOT USED dL = [0.1 0 0]; %predicted distance between cp and cg kilometerse = 2.3390e-005; %drag properties Cd = 2.2; %drag coeff corresponding to shape Kaero = −0.5*Cd*V^2; Psolar = 2*psun; %constants for aero and solar torque calculation density; %Spacecraft Magnetic Properties (assumed) mresid = [0 0 0.001]; %Spacecraft residual magnetic moment M = mresid; %Magnetic unit dipole vector K = 7.943e15; % ACTUAL Spacecraft Inertia conditions Ix = 16.67; Iy = 16.67; Iz = 16.67; Ixy = 0; Iyz = 0; Ixz = 0; Imo = [Ix −Ixy −Ixz; −Ixy Iy −Iyz; −Ixz −Iyz Iz]; %Moment of inertia matrix Iinv = inv(Imo); %Moment of inertia inverse goes in dynamics block %Spacecraft initial Euler state angles and rates phio = 0; thetao = 0; psio = 0; %Initial Euler Angles phidoto = 0; thetadoto = 0; psidoto = 0; %Initial Euler Rates %Calculation of initial quaternion (qo) and angular momentum (Ho) s1 = sin(phio/2); s2 = sin(thetao/2); s3 = sin(psio/2); c1 = cos(phio/2); c2 = cos(thetao/2); c3 = cos(psio/2); q1o = s1*c2*c3-c1*s2*s3; q2o = c1*s2*c3 + s1*c2*s3; %Wie pg. 321 q3o = c1*c2*s3-s1*s2*c3; q4o = c1*c2*c3 + s1*s2*s3; S1 = sin(phio); S2 = sin(thetao); S3 = sin(psio); C1 = cos(phio); C2 = cos(thetao); C3 = cos(psio); wxo = phidoto − psidoto*S2 − wo*S3*C2; wyo = thetadoto*C1 + psidoto*C2*S1 − wo*(C3*C1 + S3*S2*S1); wzo = psidoto*C2*C1 − thetadoto*S1 − wo*(S3*S2*C1-C3*S1); qo = [q1o q2o q3o q4o]; Ho = Imo*[wxo wyo wzo]’; norm(Ho)*1000; %Calculate eclipse time for comparison with EPS calculations Te = 100.87*2*V/2/pi; %CMG Properties (in degrees) %beta = 90*pi/180; %Skew angle in degrees converted to radians beta = [90; 90; −90; 0]; beta = beta.*pi./180; %beta = [54.73; 54.73; −54.73; 0]; beta = beta.*pi./180; Gimbal0 = [−30*pi/180; 90*pi/180; −30*pi/180; 0]; % Initial Gimbal angles for 0 H spin up w_wheel = 2800*(2*pi/60); %Wheel speed in RPM converted to rad/s Iwheel = 0.0614*1.3558179483314; % Wheel inertia in slug-ft^2 converted (exact) to kilogram m^2 h_wheel = Iwheel*w_wheel; % CMG Wheel Angular Momentum %FEEDFORWARD (ASSUMED) Spacecraft Inertia conditions Ix = 16.67; Iy = 16.67; Iz = 16.67; Ixy = 0; Iyz = 0; Ixz = 0; Imo = [Ix −Ixy −Ixz; −Ixy Iy −Iyz; −Ixz −Iyz Iz]; %Moment of inertia matrix THETAo = [Ix Ixy Ixz Iy Iyz Iz 0 0 0]; % [Fossen]’s adaptive feedforward parameters ETA = 1; LAMBDA = 0; uffGain = 1; %FEEDBACK CONTROLLERS Follow %PDI Controller Gains % TUNED Well for Presence of LOWPASS (Kp = 0.5; Kd = Kp*750; Ki = 0.1) %Kp = 0.5; Kd = Kp*750; Ki = 0.1; %PDI Controller Gains % TUNED WELL FOR NO-NOISE (Kp = 1; Kd = 2000*Kp; Ki = 5) Kp = 1; Kd = Kp*6000; Ki = 5; %PDI Controller Gains % TUNED WELL FOR NOISE (Kp = 1; Kd = Kp*3000; Ki = 1) %Kp = 1; %Kd = Kp*3000; %Ki = 1; %PID Controller Gains tuned well for uff and ufb decoupling Kpx = 20; Kdx = 1000; Kix = 0.1; %Bandpass filter pole per Bong Wie 2nd Edition pg 137–138 wp = 10*pi/SlewTime; % Product of pole frequency and zero frequency establishes max phase lag wz = 2*wp; dampZ = 1; % >0 but small for good tracking. dampP = dampZ; % Sensor Noise Parameters NoiseVariance = 1e-9; BADNoiseVariance = NoiseVariance*1e3; % Observer Gains MaxI = 100; lambda1 = 12.5; lambda2 = 50; lambda3 = 200; Kdo = MaxI*(lambda1 + lambda2 + lambda3)/10; Kpo = MaxI*(lambda1*(lambda2 + lambda3) + lambda2*lambda3)/10; Kio = MaxI*lambda1*lambda2*lambda3/9; m = 100*eye(3); minv = pinv(m); x0 = [R; 0; 0]; xdot0 = cross([0; 0; wo], x0) %gravity = −mu/(R^2); orbitperiod = 2*pi*sqrt((R^3)/mu);

**Algorithm A2.** StopFcn callbacks for SIMULINK model depicted in Figure 3, Figure 4, Figure 5 and Figure 6
%% Means and Standard Deviations for solvers quaternion format long load(‘qneuler.mat’); load(‘qnheun.mat’); load(‘qnrk.mat’); load(‘qnode5.mat’); load(‘qnrk50.mat’); load(‘qnrk100.mat’); load(‘qnrk500.mat’); qnorm = [qneuler(2,:); qnheun(2,:); qnrk(2,:); qnode5(2,:)]; QNORMSTD = zeros(1,4); for i = 1:4 QNORMSTD(i) = std(qnorm(i,:)); QNORMSTDSTRING = num2str(QNORMSTD); QnormLEGEND = [‘||q||=’, QNORMSTDSTRING]; end QNORMSTD; figure; plot(qnrk50(1,:),qnrk50(2,:),‘k--’,‘linewidth’,1.5); hold on; qnorm = std(qnrk50(2,:));QNORMSTDSTRING = num2str(qnorm); Q50 = [‘ ||q_{50}||=’, QNORMSTDSTRING]; plot(qnrk100(1,:),qnrk100(2,:),‘g:’,‘linewidth’,1.5); hold on; qnorm = std(qnrk100(2,:));QNORMSTDSTRING = num2str(qnorm); Q100 = [‘||q_{250}||=’, QNORMSTDSTRING]; plot(qnrk500(1,:),qnrk500(2,:),‘r-.’,‘linewidth’,1.5); hold on; qnorm = std(qnrk500(2,:));QNORMSTDSTRING = num2str(qnorm); Q500 = [‘||q_{500}||=’, QNORMSTDSTRING]; plot(qnrk(1,:),qnrk(2,:),‘b’,‘linewidth’,1.5); hold on; qnorm = std(qnrk(2,:));QNORMSTDSTRING = num2str(qnorm); Q1000 = [‘||q_{1000}||=’, QNORMSTDSTRING]; legend([Q50],[Q100],[Q500],[Q1000],‘FontName’,‘Palatino Linotype’,‘FontSize’,10,‘location’,‘best’); xlabel(‘Time (s)’,‘FontName’,‘Palatino Linotype’,‘FontSize’,10) set(gca,‘FontName’,‘Palatino Linotype’,‘FontSize’,10); grid on; set(gcf,‘units’,‘inches’,‘Position’,[1 1 7 2.5]) %% 3D Trajectory and altitude change over orbits load(‘long300.mat’) subplot(2,1,1); plot3(long300(2,:),long300(3,:),long300(4,:),‘Linewidth’,1.5); zlim([-175 175]); hold on; grid on subplot(2,1,2); plot(long300(1,:),long300(6,:)-300e3,‘Linewidth’,1.5); ylim([−80 350]); grid on; xlabel({‘Time (s)’},‘FontName’,‘Palatino Linotype’,‘FontSize’,10), ylabel({‘\Delta h (m)’},‘FontName’,‘Palatino Linotype’,‘FontSize’,10); set(gca,‘FontName’,‘Palatino Linotype’,‘FontSize’,10); set(gcf,‘units’,‘inches’,‘Position’,[1 1 7 3]) %% Altitude change over single orbit—with and without tether load(‘rise200.mat’); load(‘rise250.mat’); load(‘rise300.mat’); load(‘rise350.mat’); load(‘rise500.mat’); load(‘fall200.mat’); load(‘fall250.mat’); load(‘fall300.mat’); load(‘fall350.mat’); load(‘fall500.mat’); figure; subplot(1,2,1); plot(fall200(1,:),fall200(6,:)-200e3,‘-.’,fall250(1,:),fall250(6,:)-250e3,‘--’,fall300(1,:),fall300(6,:)-300e3,fall350(1,:),fall350(6,:)-350e3,‘:’,fall500(1,:),fall500(6,:)-500e3,‘--’,‘LineWidth’,1.5); grid on; ylim([−1000 1000]); xlabel({‘Time (s)’; ‘(a)’},‘FontName’,‘Palatino Linotype’,‘FontSize’,10), ylabel({‘\Delta h (m)’},‘FontName’,‘Palatino Linotype’,‘FontSize’,10); legend(‘200 kilometers’,‘250 kilometers’,‘300 kilometers’,‘350 kilometers’,‘500 kilometers’,‘location’,‘best’); set(gca,‘FontName’,‘Palatino Linotype’,‘FontSize’,10); subplot(1,2,2); plot(rise200(1,:),rise200(6,:)-200e3,‘-.’,rise250(1,:),rise250(6,:)-250e3,‘--’,rise300(1,:),rise300(6,:)-300e3,rise350(1,:),rise350(6,:)-350e3,‘:’,rise500(1,:),rise500(6,:)-500e3,‘--’,‘LineWidth’,1.5); grid on; ylim([−1000 1000]); xlabel({‘Time (s)’; ‘(a)’},‘FontName’,‘Palatino Linotype’,‘FontSize’,10), ylabel({‘\Delta h (m)’},‘FontName’,‘Palatino Linotype’,‘FontSize’,10); %legend(‘200 kilometers’,‘250 kilometers’,‘300 kilometers’,‘350 kilometers’,‘500 kilometers’,‘location’,‘best’); set(gca,‘FontName’,‘Palatino Linotype’,‘FontSize’,10); set(gcf,‘units’,‘inches’,‘Position’,[1 1 7 3]) %% Numerical Values for Spacecraft Altitude %DATA REQUIRED: all files from prev format long; at200 = [rise200(1,end), fall200(6,end), rise200(6,end), rise200(6,end)- fall200(6,end)] at250 = [rise250(1,end), fall250(6,end), rise250(6,end), rise250(6,end)- fall250(6,end)] at300 = [rise300(1,end), fall300(6,end), rise300(6,end), rise300(6,end)- fall300(6,end)] at350 = [rise350(1,end), fall350(6,end), rise350(6,end), rise350(6,end)- fall350(6,end)] at500 = [rise500(1,end), fall500(6,end), rise500(6,end), rise500(6,end)- fall500(6,end)] %% Lorentz force extracted from tether—altitude and inclination load(‘inc0.mat’); load(‘inc10.mat’); load(‘inc30.mat’); load(‘inc60.mat’); load(‘inc90.mat’); figure subplot(1,2,1); plot(rise200(1,:),rise200(7,:),‘-.’,rise250(1,:),rise250(7,:),‘--’,rise300(1,:),rise300(7,:),rise350(1,:),rise350(7,:),‘:’,rise500(1,:),rise500(7,:),‘--’,‘LineWidth’,1.5); grid on; ylim([0 0.02]); xlabel({‘Time (s)’; ‘(b)’},‘FontName’,‘Palatino Linotype’,‘FontSize’,10), ylabel({‘\Delta h (m)’},‘FontName’,‘Palatino Linotype’,‘FontSize’,10); legend(‘200 kilometers’,‘250 kilometers’,‘300 kilometers’,‘350 kilometers’,‘500 kilometers’,‘location’,‘best’); set(gca,‘FontName’,‘Palatino Linotype’,‘FontSize’,10); subplot(1,2,2); plot(inc0(1,:),inc0(7,:),‘-.’,inc10(1,:),inc10(2,:),‘--’,inc30(1,:),inc30(2,:),inc60(1,:),inc60(2,:),‘:’,inc90(1,:),inc90(2,:),‘--’,‘LineWidth’,1.5); grid on; ylim([0 0.02]); xlabel({‘Time (s)’; ‘(b)’},‘FontName’,‘Palatino Linotype’,‘FontSize’,10), ylabel({‘Force (N)’},‘FontName’,‘Palatino Linotype’,‘FontSize’,10); legend(‘0’,‘10’,‘30’,‘60’,‘90’,‘location’,‘best’,‘NumColumns’,2); set(gca,‘FontName’,‘Palatino Linotype’,‘FontSize’,10); set(gcf,‘units’,‘inches’,‘Position’,[1 1 7 3]) %% Lorentz force means and standard deviations varying inclinations m0 = mean(inc0(7,:)) m10 = mean(inc10(2,:)) m30 = mean(inc30(2,:)) m60 = mean(inc60(2,:)) m90 = mean(inc90(2,:)) sd0 = std(inc0(7,:)) sd10 = std(inc10(2,:)) sd30 = std(inc30(2,:)) sd60 = std(inc60(2,:)) sd90 = std(inc90(2,:)) %% Deflection 3D plot load(‘def0.mat’); load(‘def10.mat’); load(‘def20.mat’); figure; plot3(def0(2,:),def0(3,:),def0(4,:),‘Linewidth’,1.5); hold on; plot3(def10(2,:),def10(3,:),def10(4,:),‘Linewidth’,1.5); hold on; plot3(def20(2,:),def20(3,:),def20(4,:),‘Linewidth’,1.5); hold on; legend(‘0’,‘10’,‘20’,‘location’,‘best’); view(−45,15); set(gca,‘FontName’,‘Palatino Linotype’,‘FontSize’,10); set(gcf,‘units’,‘inches’,‘Position’,[1 1 7 2]); grid on %xlim([0 400]); ylim([−200 20]); zlim([−1 2]); view(−45,15);
